# Investigation of synergistic effects incorporating esterified lignin and guar gum composite aerogel for sustained oil spill cleanup

**DOI:** 10.1038/s41598-024-64623-2

**Published:** 2024-06-17

**Authors:** Mahnaz Montazeri, Reza Norouzbeigi

**Affiliations:** Nanomaterials and Surface Technology Research Laboratory, School of Chemical, Petroleum and Gas Engineering, Iran University of Science and Technology, Narmak, P. B. 16765-163, Tehran, Iran

**Keywords:** Biomass-based aerogel, Guar gum-lignin composite, Lignin esterification, Oil absorption, Chemistry, Materials science, Nanoscience and technology

## Abstract

The recently developed aerogel demonstrates a high capacity for pollutant absorption, making it an environmentally friendly option for oily water treatment. In an effort to reduce the adverse effects of the black liquor accumulation in the pulp industry, this study focused on utilizing the mentioned abundant bio-resource lignin, which can be applied to various high-value applications such as 3D porous materials for oil spill cleanup. Lignin, precipitated from the black liquor, was esterified using maleic anhydride as the esterifying reagent to enhance the hydrophobicity. Then, the composite aerogel fabricated from esterified lignin and guar gum (GG) was successfully prepared through the facile freeze-drying, using glutaraldehyde (GA) as the cross-linker. The resulting aerogel exhibited high porosity values exceeding 95%, low density (27.4 mg/cm^3^), and an impressive absorption capacity of 32.5 g/g for sunflower oil. These results demonstrate the potential of black liquor utilization as a bio-waste source of lignin and highlight the cost-effective guar gum-esterified lignin composite aerogel, which exhibits remarkable oil absorption capabilities and environmental sustainability promotion.

## Introduction

The lack of clean water is a consequence of rapid development and industrial policies, which have had adverse effects on water quality and raised environmental concerns. over the years, researchers have concentrated emphasis on eliminating, or at least reducing, organic and inorganic pollutants from waste water, particularly from industrial effluents^1^. A variety of methods have been suggested and classified into physical, chemical, mechanical, and biological approaches for cleaning oily wastewater. Instead, the limitations of these traditional methods are their time-consuming, secondary pollution, and costly processes^2,3^.

Porous materials are extensively employed for absorption purposes owing to their notable efficacy, cost-effectiveness, user-friendliness, and limited secondary contamination to the environment^4^. Various porous superhydrophobic materials with super-oleophilic properties have been regarded as desirable options for oil spill cleanup. so far, numerous researchers have investigated the utilization and application of innovative sorbents for removal of the water contaminants^5^. Recently, significant studies have been concentrated on the development of sustainable natural three-dimensional porous materials for oil removal such as superhydrophobic/superoleophilic foams^6^, sponges^7^, and aerogels^8^.

Aerogels are highly porous materials produced by displacing the liquid solvent in a gel with air while concurrently maintaining a three-dimensional (3D) network structure. These materials possess a multitude of applications including, the removal of heavy metal ions and organic dyes, cleanup of oil spills, and more, owing to their high porosity, extremely low density, and larger surface area^9^. However, several challenges need to be overcome during the preparation of aerogels, including high manufacturing costs, nono-biodegradable precursors, and low mechanical strength, which may decrease their suitability for commercialization^10^.

Biomass-derived aerogels obtained from natural organic sources are widely recognized as desired choices for oil absorption considering their abundance, low cost, renewability, biodegradability, and almost facile surface chemical modifications^8^. For example, Yan et al. developed aerogels by freeze-drying a suspension with low bacterial cellulose (BC) concentration, followed by a chemical vapor deposition modification with methyltrimethoxysilane as the modifier and ammonia as the catalyst. The aerogels reveal simultaneously high absorption capacity (121.8–284.1 g/g) and excellent recyclability^11^. Guo et al. reported that the reduced graphene oxide (rGO)/chitosan (CS) aerogel microspheres demonstrate a highly porous network and a center-diverging microchannel structure. It can also separate surfactant-stabilized water-in-oil and oil-in-water emulsions through demulsification^12^.

Lignin exhibits high levels of adaptability as a favorable precursor for fabrication of bio-based aerogels due to its approachability, multitude of available surface functional groups, and cost-effectiveness^8,13^. For example, Yue et al. fabricated a lignin-mediated fire-resistant aerogel for repeated oil/water separation. The introduction of lignin not only enables the high-value utilization of lignin but also leads to a porous structure and low density, enhancing the hydrophobicity and thermal stability of rGO^14^. Chen et al. synthesized Hydroxyethyl cellulose-lignin aerogel (CL-aerogel) by combining hydroxyethyl cellulose with lignin using the sol–gel method followed by freeze-drying. The incorporation of lignin endowed the aerogel with a 3D porous network and enhanced mechanical properties. Then CL-aerogel was modified with n-dodecyl mercaptan (NDM) and Fe_3_O_4_ nanoparticles through ultrasound enhancement to obtain HMCL-aerogel. Meanwhile, the aerogel exhibited effective separation of oil/water mixture with flux as high as 2986 L/m^2^/h even in corrosive conditions and maintained over 99% separation efficiency after 10 separation cycles^15^.

Furthermore, lignin, sometimes known as black liquor, is primarily produced as a byproduct of pulp industry (accumulated ~ 70 million tons/year worldwide), with the majority being used as fuel. Considering the requirements and basics of the sustainability, conversion of these massive by-products into the value-added outcomes (such as 3D porous materials) for different applications including medical, oil/water separation, energy storage, and etc., is reasonably beneficial^16^.

In order to enhance the lignin applicability, chemical modification processes can often be conducted. The main building blocks of lignin consist of *p*-hydroxyphenyl, guaiacyl, and syringyl aromatic repeating units, which are interconnected through either aryl ether or carbon–carbon linkages^17^. The abundant presence of aliphatic and aromatic hydroxyl groups in lignin has been effectively utilized for modifying its structure. As an example, esterification can replace the hydroxyl groups in lignin with ester substituents, thereby enhancing its hydrophobicity, which is crucial for improving the selectivity and efficiency of the oil absorption processes^18,19^.

Guar gum (GG) is an essential hydrocolloid polysaccharide employed in diverse commercial applications, consisting of linearly bonded mannose units with alternating lateral branched galactose groups. The water solubility, viscosity, abundance, and cost of GG render it exceptionally appealing^20,21^.

The combination of these two natural polymers has the gain of the aerogel applicability enhancement and provide valuable insights into the characterization and oil absorption behavior of the resulted aerogels. Therefore, the aim of this study was fabrication of an effective and stable aerogel complex containing guar gum and esterified lignin derived from the black liquor resulted from the pulp industry. The aerogels were prepared by mixing esterified lignin with guar gum in the presence of glutaraldehyde (as cross-linker), followed by gelation, aging, and subsequently the freeze-drying. All the prepared aerogels were evaluated for their physical properties, thermal stability, and surface morphology. Furthermore, surface modification was applied in order to hydrophobicity achievement, and accordingly the water contact angles and oil absorption efficiencies were determined.

## Experimental procedure

### Materials

Guar gum (GG), acetone (C_3_H_6_O), sulfuric acid (H_2_SO_4_,98%), glutaraldehyde, maleic anhydride, toluene (C_6_H_5_CH_3_), and Octadecyl trichlorosilane (ODTS) (CH_3_(CH_2_)_17_SiCl_3_) were purchased from Merck. Softwood black-liquor was provided by Iran pulp mill Manufacturing.

### Extraction and esterification of lignin

Lignin was obtained using a procedure optimized for maximizing lignin precipitation yield, as described in our previous work^22^. Initially, to ensure the precise temperature control, a black liquor solution (100 ml, 38.5% solid content) was placed in a water bath. Subsequently, the black liquor was precipitated at 75 °C under constant stirring by slowly addition of sulfuric acid solution (72%wt) until pH value reached to three. The resulting slurry was continuously stirred for an appropriate aging time (120 min) to allow the reaction completion and formation of the precipitates. The precipitates were then filtered, washed (multiple times) with distilled water to remove excess acid, and dried in an oven (70 °C, 24 h.).

The esterification of the lignin was performed using maleic anhydride. A total of 10 g of the precipitated lignin was added to a solution of maleic anhydride (10%) in acetone at a ratio of 1:20 (w/v)^23^. Subsequently, the mixture was continuously stirred for 6 h at 60 °C in a reflux system. Once the reaction was completed, the mixture was allowed to remain at room temperature for 24 h to facilitate the acetone evaporation. Finally, the esterified lignin (EL) was filtered and washed by hot distilled water to eliminate any unreacted maleic anhydride and by-products, and then oven-dried at 70 °C.

### Preparation of the guar gum/esterified lignin aerogel

**Figure 1:**
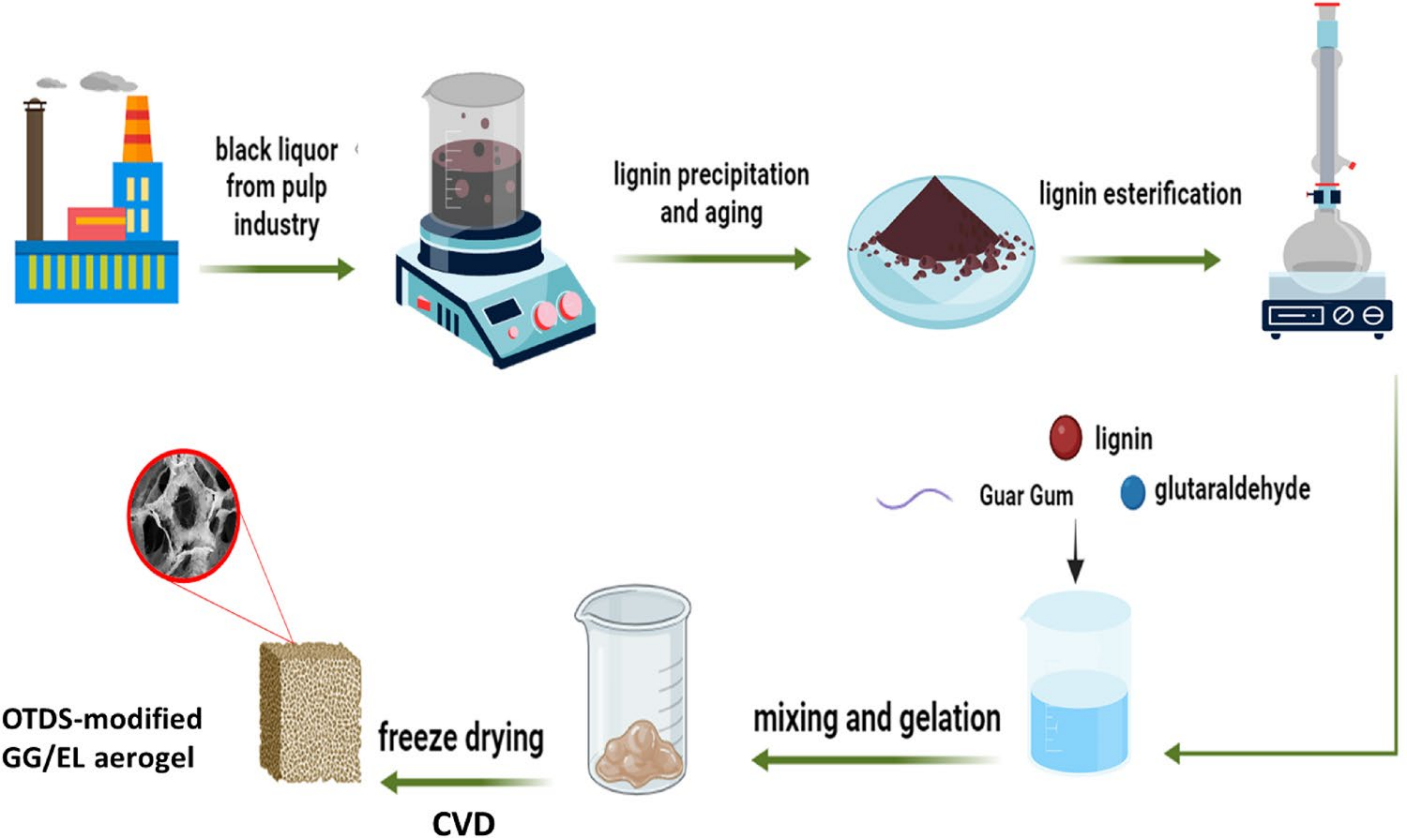
The schematic diagram of lignin precipitation from black liquor and fabrication of OTDS-modified GG/EL aerogel.

The guar gum/esterified lignin aerogel was prepared by mixing appropriate amounts of EL in the guar gum (GG) solution and adding glutaraldehyde as cross-linking agent. Initially, the guar gum solution was prepared by adding 0.5 g of guar gum to 50 ml of distilled water and dispersing the mixture through overnight stirring. Then, 0.5 g of EL was mechanically stirred into the guar gum solution to create a uniform suspension, which was subsequently dispersed for 1 h using an ultrasonic bath. After this step, glutaraldehyde (1 ml) was added dropwise to the previously prepared mixture using a mechanical stirrer for 30 min, and the resulting mixture was left undisturbed for another 4 h. The obtained gel was poured into a mold, frozen at − 80 °C, and then subjected to freeze dryer at − 60 °C under vacuum for 48 h. till the GG/EL aerogel (4*4*1.5 cm) fabrication. For the silanization reaction, the GG/EL aerogel and a small open beaker containing 1 ml of octadecyltrichlorosilane (OTDS) were placed in a large sealed container and heated in an oven at 120 °C for three hours. Figure 1 illustrates the procedure for preparing OTDS-modified GG/EL composite aerogel.

### Characterization of GG/EL aerogel

The surface morphology of the GG/EL aerogels was examined using a scanning electron microscope (SEM, Tuscan, model VEGA2) operating at 30.0 kV. The functional groups present in the samples were determined using an FTIR spectrophotometer (ATR-FTIR, model ATR-8000, Shimadzu, Japan) in the range of 400–4000 cm^−1^. The hydrophobicity of the OTDS-modified GG/EL aerogels was evaluated using a contact angle-measuring device (OCA 20, Data Physics ES, Germany) at room temperature. The esterified lignin achieved from black liquor was characterized utilizing ^1^H NMR analysis on a Varian INOVA 500 MHz NMR instrument, with DMSO-d6 as the NMR solvent at 18 °C. Thermal stability analysis of the products was performed using a classical thermogravimetric analysis (TGA, Thermal instrument STA 409 PC LuxxÒ manufactured by NETZSCH-Germany) with a heating rate of 10 °C/min up to 700 °C.

### Density and porosity measurements

The common properties of the prepared aerogels, such as their porosity and density, were examined. The apparent density of the GG/ EL aerogel (ρa) was obtained through dividing the weight of the GG/EL aerogel by its volume and the bulk density of GG/ EL (ρs) aerogel was assessed with the formula (1) and the porosity of fabricated aerogel was calculated by the following formula (2)^24^.

1 $${\rho }_{s}=\frac{1}{\frac{{w}_{EL}}{{\rho }_{EL}}+\frac{{w}_{GG}}{{\rho }_{GG}}}$$

2 $$Porosity(\%)=\left(1-\frac{{\rho }_{a}}{{\rho }_{s}}\right)\times 100$$

where W_lignin_ is the weight fraction of the esterified lignin and the W_SA_ is the weight fraction of GG.

### Absorption test

In order to investigate the oil absorption capacity of GG/EL aerogel, the fabricated aerogel was immersed in sunflower oil for 5 min then by weighing the aerogel before and after of the oil absorption, the capacity was determined by the Eq. (3):

3 $$Q=\frac{m-{m}_{0}}{{m}_{0}}$$

where m_0_ and m represent the mass of the GG/EL aerogel before and after saturated oil absorption, respectively. The reusability of the prepared aerogel for continuously repeated utilization cycles was also examined by applying the common absorption-squeeze approach.

## Results and discussion

### Properties, morphology, and surface features of GG/EL aerogels

**Figure 2:**
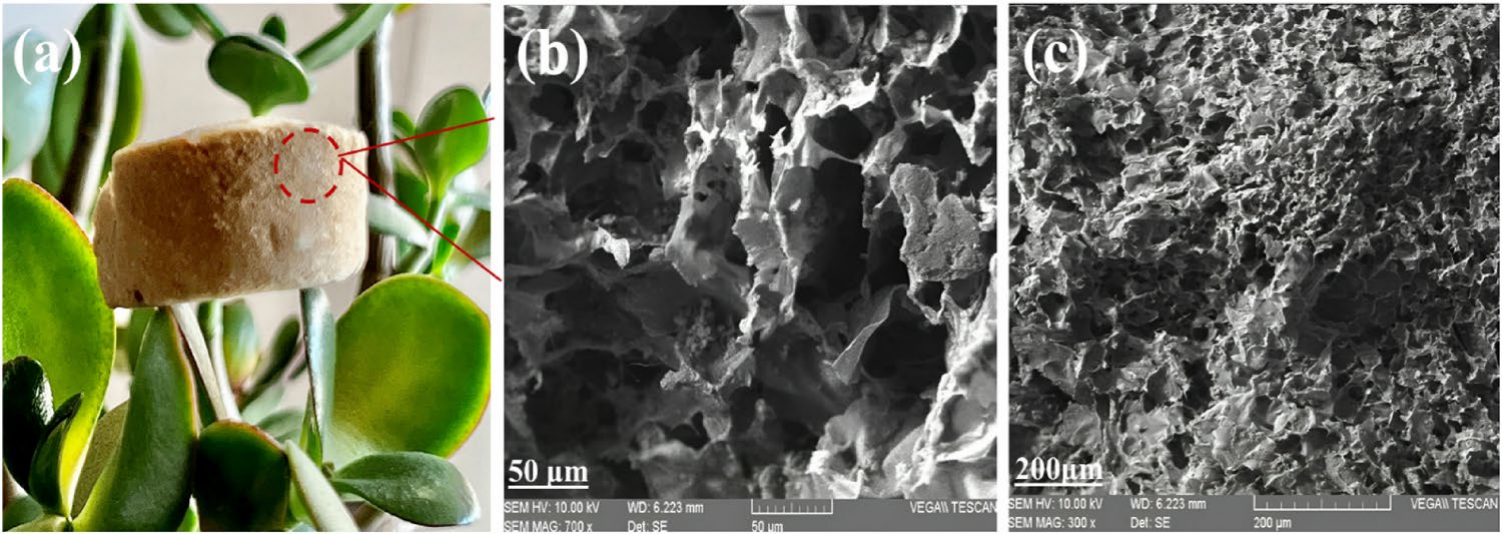
(**a**) Digital photos of the GG/EL aerogel, (**b**, **c**) SEM images of GG/EL aerogel with different magnifications (50×, 200×).

The GG/EL aerogel, prepared through freeze drying, exhibited a porous and spongy appearance with an extremely low weight, as shown in Fig. 2a. The surface morphology of the GG/EL aerogel was investigated by SEM, as depicted in Fig. 2b,c. The results revealed that all GG/EL aerogels typically comprise an interconnected 3D porous structure with distinct pores, primarily attributed to the sublimation of water during the freeze-drying process, which is desirable for storing the absorbed oil^25^. The GG/EL aerogel demonstrates an extensive range of pore sizes and numerous macro-pores as a result of the extensive cross-linking with glutaraldehyde. Crosslinking plays a crucial role in enhancing the stability of aerogels by strengthening the structural integrity of the aerogel network, preventing collapse or deformation of the porous structure. Additionally, crosslinking can improve the mechanical strength and resistance to degradation of aerogels, thereby making them more robust and durable^26^.

The crosslinking process between guar gum and glutaraldehyde occurs through the reaction between the hydroxyl groups of guar gum and the aldehyde groups of glutaraldehyde. Glutaraldehyde is a bifunctional molecule with two aldehyde groups at each end, enabling it to form covalent bonds with hydroxyl groups^27,28^.

Without the presence of a cross-linking agent, the pure guar gum aerogel is prone to shrinkage and collapse, resulting in the loss of its structural integrity^29^. The crosslinking agent glutaraldehyde can effectively prevent the aerogel from shrinkage and helps the durability of the aerogel structural integrity. The GG/EL aerogel reveled high porosity values (> 95%), and its density was about 27.4 mg/cm^3^. According to BET analysis results, the synthesized Guar gum/esterified lignin aerogel shows a low surface area (about to 36.38 m^2^/g).

### FTIR analysis

**Figure 3:**
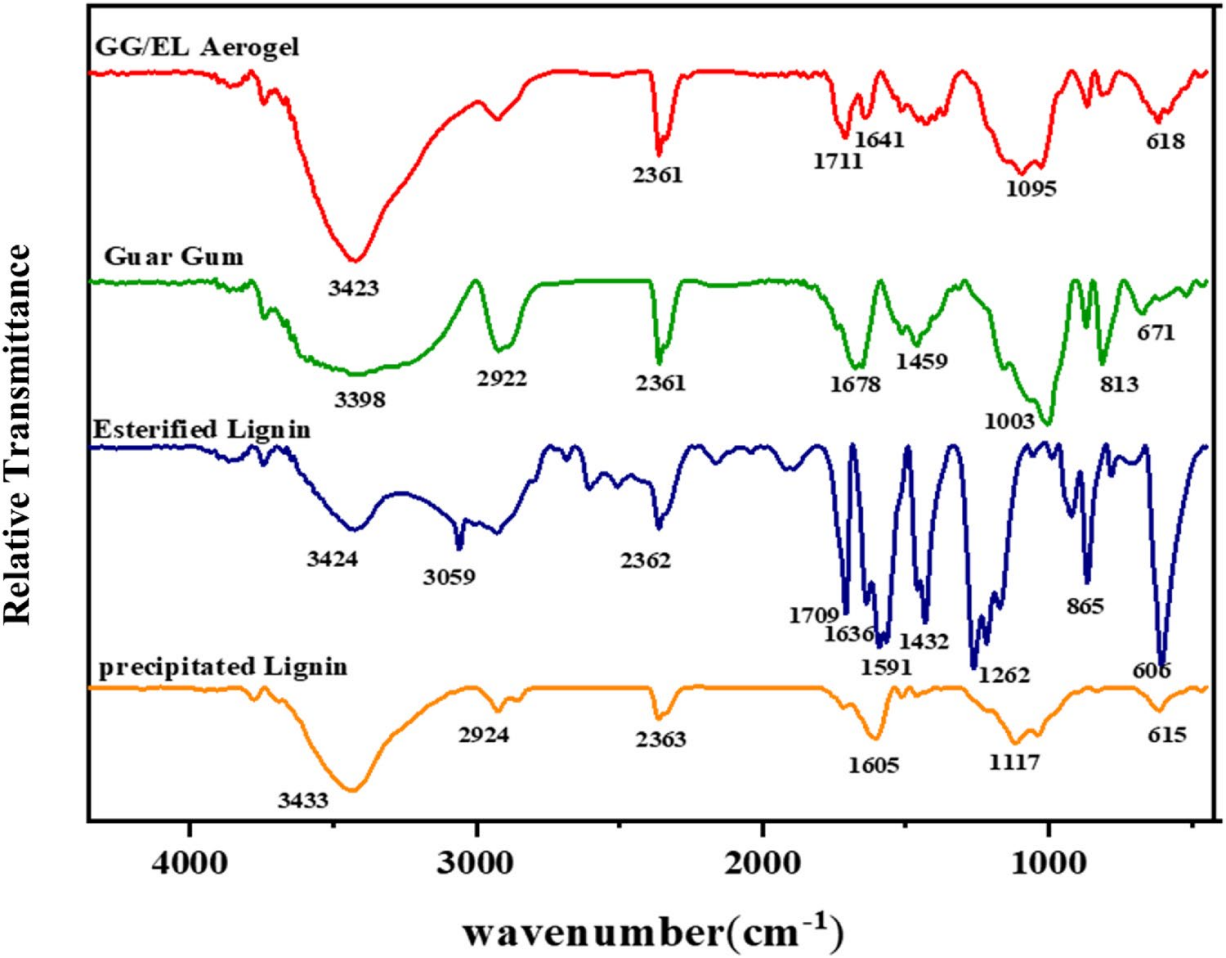
FTIR spectra of the precipitated and esterified lignins, guar gum, and GG/EL aerogel.

The FTIR spectra of the modified and unmodified lignin samples are presented in Fig. 3. As depicted in Fig. 3, the FTIR spectra demonstrate the changes in the primary functional groups resulting from the esterification of the precipitated lignin from black liquor into esterified lignin. The unmodified precipitated lignin exhibits a strong broad peak at 3424 cm^−1^, which corresponds to the hydroxyl groups stretching in aliphatic and phenolic structures. Esterification reactions with maleic anhydride lead to a decrease in the hydroxyl groups existing in the precipitated lignin. Furthermore, the observed notable absorption bands/peaks at approximately 1709 cm^−1^ can be related to the ester C=O group stretching and vibrations^30,31^. The appeared increase in the intensities of the absorption peaks at 1262 cm^−1^ (C–O) confirms the mentioned point. The stretching vibration at 1636 cm^−1^ is typically indicative of the presence of a carbonyl group (C=O) in the modified lignin molecule, which can be introduced through esterification reactions^32^. Furthermore, peaks placed around 1591 cm^−1^ and 1427 cm^−1^ correspond to the C–C stretching of the aromatic ring, and these peaks have been also enhanced by esterification^23^.

The presence of hydroxyl groups in guar gum is typically identified by a broad peak in the range of 3200–3600 cm^−1^, which corresponds to the stretching vibration of the O–H bond^33^. In the case of guar gum, significant peaks can be detected at 3600–3200 cm^−1^ (stretching vibration of O–H) and 2922 cm^−1^ (stretching vibration C–H)^34^. The presence of glycosidic linkages in guar gum, which play a crucial role in its polysaccharide structure, can be observed within the range of 1000–1200 cm^−1^. These peaks correspond to the stretching vibrations of the C–O–C bonds present in the glycosidic linkages^33^. In the spectrum of GG/EL aerogel additional peaks can be observed around 1700–1800 cm^−1^ could suggest the presence of a carbonyl group, which is formed when the aldehyde groups of glutaraldehyde react with the hydroxyl groups of guar gum^35,36^.

### ^1^H NMR spectra of esterified lignin

**Figure 4:**
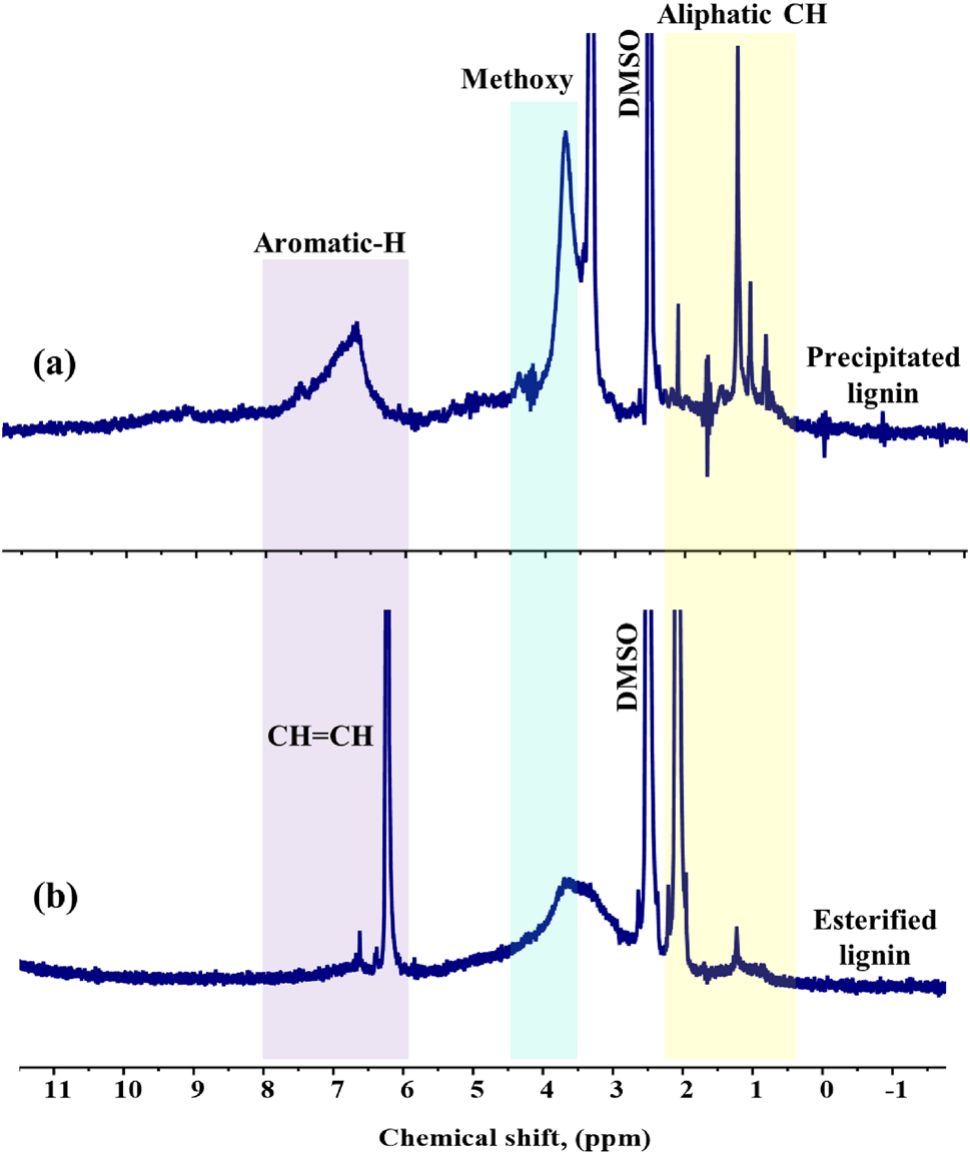
^1^H NMR spectra of (**a**) precipitated lignin and (**b**) esterified lignin.

Precipitated and esterified lignins have been well-characterized by ^1^H NMR (Fig. 4a,b). For the ^1^H NMR spectrum of precipitated lignin, the characteristic peaks at 6.3–8.4, 3–4, and 0.5–2.4 ppm can be attributed to the aromatic ring skeleton, methoxy, and acetyl protons, respectively (Fig. 4a)^30,37^. The two most prominent differences between modified and unmodified lignin spectra can be observed. Firstly, the esterified lignin exhibited new peaks in the chemical shift region of 2–2.5 ppm, which can be attributed to the alkyl protons of the ester groups, confirming the successful esterification process. Secondly, a sharp peak around 6–6.5 ppm observed at the esterified lignin spectrum could indicate the presence of a double bond within the esterified lignin structure. On the other hand, the prominent signal observed at 6 ppm serves as a distinct indication of the presence of aliphatic protons that are directly bonded to a double bond (–HC=CH–)^38,39^.

### Thermal analysis

**Figure 5:**
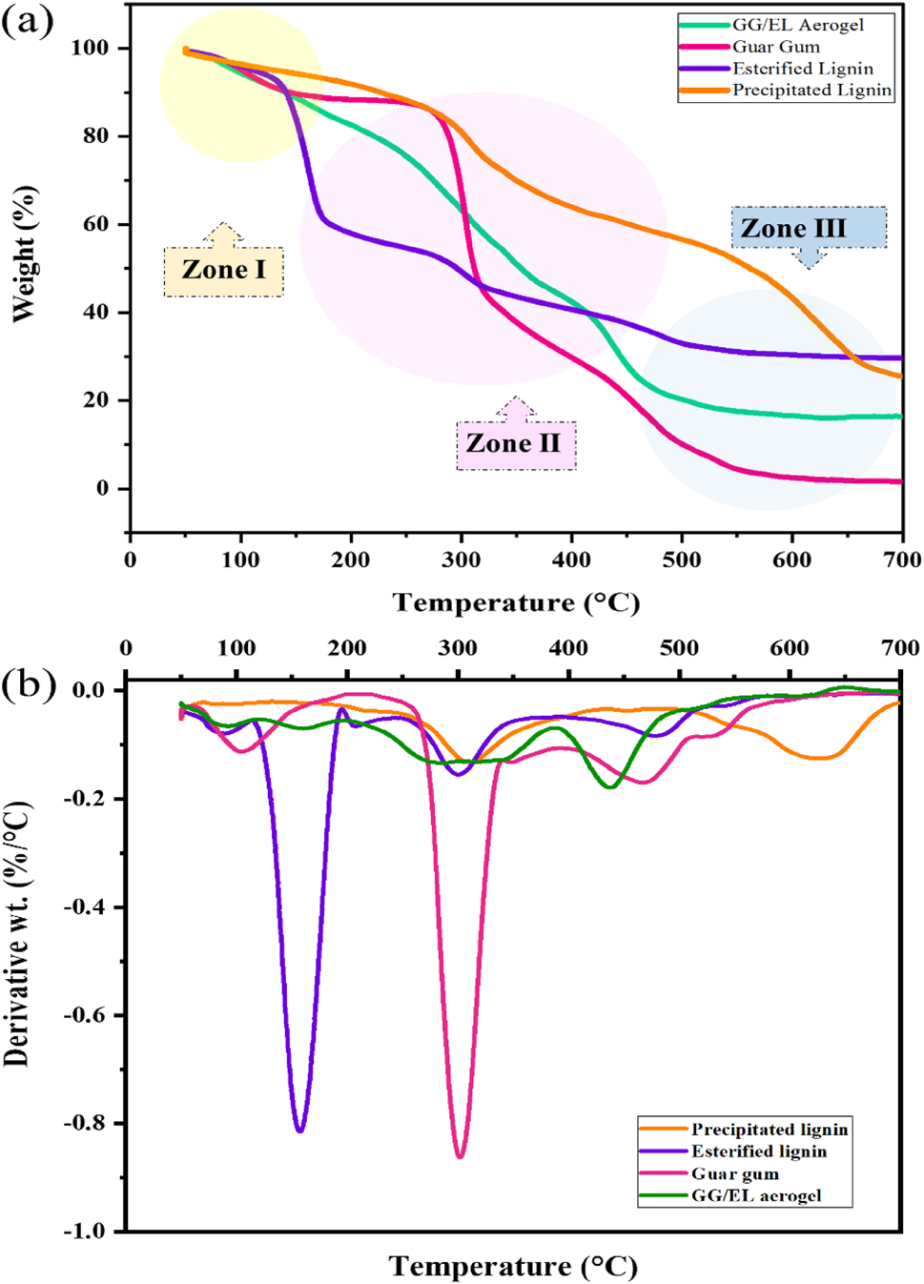
TGA (**a**) and first derivative of the TGA (**b**) curves of the precipitated lignin, esterified lignin, guar gum, and GG/EL aerogel aerogels.

The thermal behaviors and stabilities of the precipitated lignin, esterified lignin, and GG/EL aerogel were characterized by employing thermos-gravimetric analysis (TGA). The TGA curves illustrate the weight loss of the samples as a function of the temperature of thermal degradation, while the first derivative of the corresponding curve (DTG) demonstrates the rate of weight loss (Fig. 5). For comparison, the thermal degradation profiles of lignin and guar gum were also examined. As shown in Fig. 5a, three distinct thermal degradation zones can be observed. Lignin typically undergoes multiple stages of weight loss due to its complex structure and composition, with the primary degradation can occur over a wide temperature range. The TGA curve of lignin may exhibit an initial weight loss starting as low as approximately (50–200 °C), which is attributed to the evaporation of moisture and volatile compounds adsorbed on the surface^40^. Modified lignin displays distinct decomposition stages in comparison with unmodified lignin. In this stage, the esterified lignin prepared with maleic anhydride had distinct peak, attributed to its strong hydrophilicity resulting from the introduction of carboxyl groups and the reduction of hydroxyl groups occurred simultaneously during the esterification process^41^. In general, the primary degradation step of esterified lignin commences around 250–350 °C and extends up to approximately 500–600 °C or higher. Esterified lignin demonstrates a reduced rate of weight loss compared to lignin due to the enhanced thermal stability conferred by the presence of ester functional groups^23^. This can be observed as a more gradual degradation profile or a plateau region in the TGA curve. Following the initial weight loss, both lignin and esterified lignin typically exhibit a gradual weight loss over a wide temperature range^38^. This stage corresponds to the primary degradation of the polymer and is a result of the breakdown of various chemical bonds within the material's structure. In the case of lignin, this degradation stage involves the cleavage of ether, C–C, C–O, and other bonds, resulting in the formation of smaller molecular fragments and gaseous byproducts. Esterified lignin exhibited a distinguishable decomposition peak at around 177 °C owing to the presence of unsaturated double bonds^41^. The final step involved the decomposition of the main components in the lignin esters into smaller molecules, including ethane, methane, propane, carbon dioxide, carbon monoxide, and hydrogen, etc.^42^.

Additionally, the thermal degradation profiles of esterified lignin, guar gum, and GG/EL aerogel are provided for comparison. As depicted in the figure, three distinct thermal degradation regions (1, 2, 3) are observed, which is a typical trend for such composites. This weight loss behavior is a consistent trend as the intermolecular chains of the aerogels gradually decompose over a temperature range from 25 to 800 °C. Since lignin contains C–C bonds that degrade around 300–400 °C, a similar effect is observed in the degradation behavior of the aerogels, indicating improved stability^43^. The process of cross-linking creates a three-dimensional network structure within the material, which enhances the overall thermal stability of the aerogel by providing structural integrity and resistance to thermal degradation. The cross-linker restricts the mobility of the polymer chains, and accordingly increases the material's resistance to decomposition. Additionally, the fabricated aerogel exhibited noticeable strength when compared to guar gum and esterified lignin. This indicates that the incorporation of esterified lignin into the guar gum matrix somehow enhances the thermal strength of the aerogels^44^.

### Surface wettability

**Figure 6:**
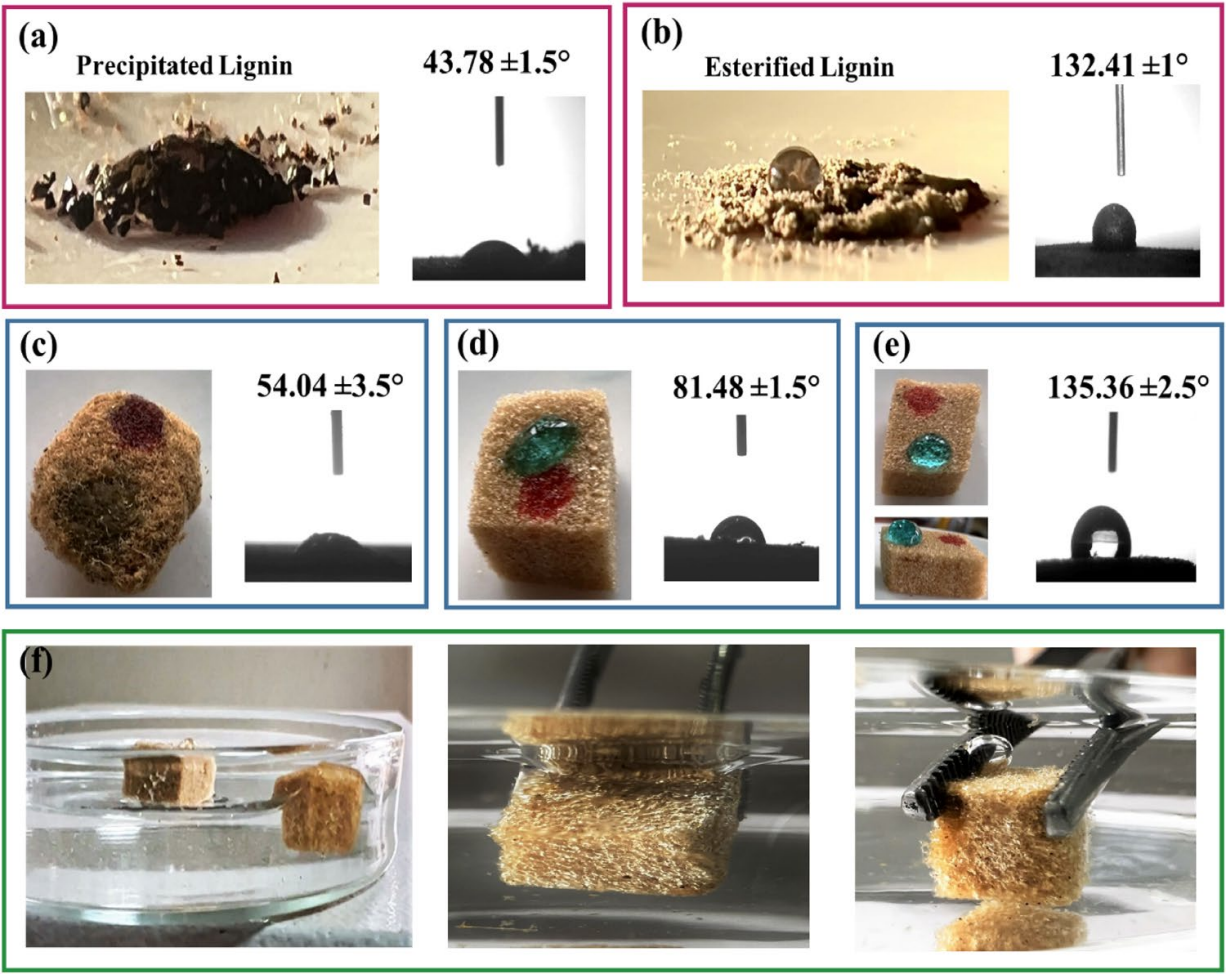
The water contact angle of (**a**) precipitated lignin, (**b**) esterified lignin, (**c**) GG/precipitated lignin aerogel, (**d**) GG/EL aerogel, (**e**) OTDS-modified GG/EL aerogel, (**f**) Digital Photos of OTDS-modified GG/EL aerogel and unmodified GG/EL aerogel when placing on water and OTDS-modified GG/EL aerogel when forcing into water.

The water-repellent properties of both precipitated and esterified lignin were evaluated through water contact angle (WCA) measurements. The water θ_CA_ values and optical photographs of water droplets on the surfaces of precipitated and esterified lignins are depicted in Fig. 6a,b. According to modification by maleic anhydride, the esterified lignin exhibited a clear increase in WCA up to 130°, demonstrating its excellent hydrophobic character. In other words, the esterification of lignin can enhance its hydrophobicity by introducing ester groups into its structure, thereby altering its chemical properties^38,45^. This process involves the replacement of hydroxyl groups in lignin with ester groups, resulting in a decrease in the number of hydroxyl groups and an increase in the number of ester groups. As a consequence, this reduction in hydrogen bonding between lignin molecules leads to hydrophobicity promotion^46^. The wettability of as-prepared aerogels is a critical property that plays a significant role in determining their suitability for oily wastewater treatment applications. Hence, water contact angle measurements were conducted for all of the prepared aerogels, including GG/precipitated lignin, GG/EL, and hydrophobic GG/EL, as depicted in Fig. 6c–e. The water contact angle of the OTDS-modified GG/EL aerogel was found to be higher than that of the two others, indicating the hydrophobic nature of the OTDS-modified GG/EL aerogel surface. Additionally, the surfaces of the aerogels were exposed to oil and water droplets, as shown in Fig. 6c. It was observed that the aerogel resulted from precipitated lignin rapidly absorbed both oil and water droplets, while the sample prepared by esterified lignin had a slightly lower absorption of water droplets compared to the third one (Fig. 6d). In the case of the OTDS-modified GG/EL aerogel, the oil droplet was absorbed completely, while the water droplet remained with a spherical shape on the aerogel (Fig. 6e). The wettability of aerogels can be influenced by modifying the roughness (topographic features) or composition (chemical aspects) of the surface. Various techniques, such as functionalization with hydrophilic or hydrophobic groups, can be employed for surface chemical modification^47^. The esterification of lignin has the benefits of the surface wettability alteration for GG/EL composite aerogels by introducing hydrophobic groups into the lignin structure and enhancing surface roughness, resulting in reduced surface wettability and increased hydrophobicity. However, the OTDS-modified GG/EL aerogel exhibits even greater hydrophobicity, which can be attributed to its low surface energy and high surface roughness. When both of the modified GG/EL aerogel and the OTDS-modified GG/EL aerogel were immersed in water simultaneously (Fig. 6f), the GG/EL aerogel absorbed water and submerged, while the modified OTDS-modified GG/EL aerogel remained buoyant for up to an hour due to its hydrophobic nature. Additionally, it can be observed that when the OTDS-modified GG/EL aerogel was subjected to external force in water, an air layer formed on its surface. Consequently, it is evident that the OTDS-modified GG/EL aerogel exhibits exceptional selective wettability, recognitions to the OTDS modification and the improvements in hydrophobicity and roughness achieved through lignin esterification. These factors play a vital role in facilitating effective oil/water separation.

### Absorption capacity of aerogel

**Figure 7:**
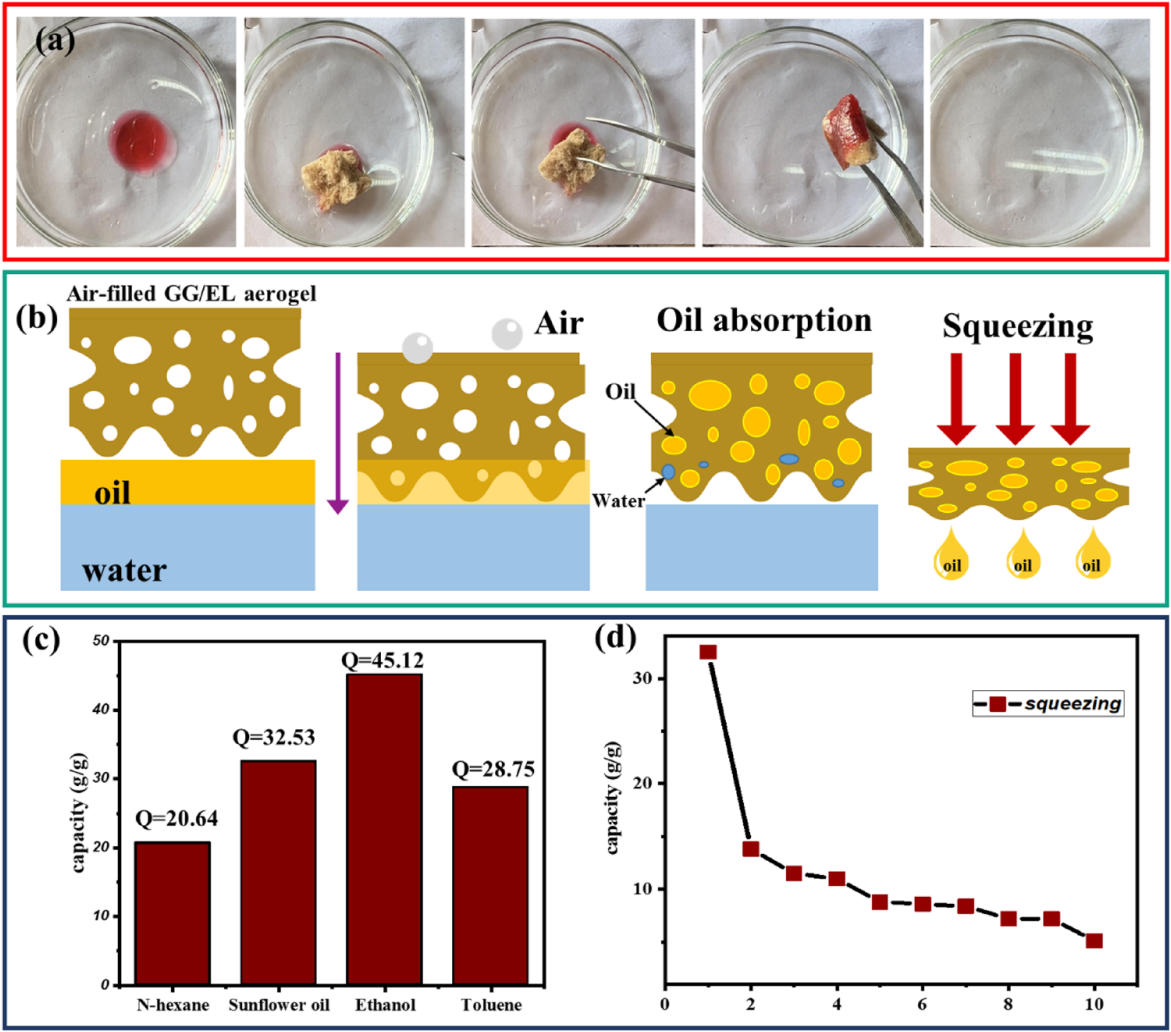
(**a**) Digital Photos of the absorption process of hydrophobic GG/EL aerogel, (**b**) a schematic procedure of oil absorption and desorption of aerogel, (**c**) Absorption capacity of the OTDS-modified GG/EL aerogel for different oils and organic solvents, (**d**) Recyclability of the OTDS-modified GG/EL aerogel done ten squeezing cycles.

The ability of OTDS-modified GG/EL aerogel to absorb oil was investigated through a qualitative experiment. Results demonstrated that the hydrophobic nature of the aerogel imposed a selective absorption of oil and organic solvents, and its porous structure contributed to a high absorption capacity. To evaluate the efficiency of OTDS-modified GG/EL aerogel oil spill cleanup, sunflower oil was used as the absorbed substance. A 25 mL sample of sunflower oil was poured into a beaker already containing water, with a small piece (0.5 g) of OTDS-modified GG/EL aerogel placed on the surface. After 5 min, the OTDS-modified GG/EL aerogel indicated the great oil-absorbing and water-repelling characteristics. Consequently, OTDS-modified GG/EL aerogel can be considered as a reliable oil absorbent for the remediation of chemical leaks or oil spills (Fig. 7a). Absorption of oil onto the OTDS-modified GG/EL aerogel occurs as a spontaneous physical process. according to the definition of aerogel, the pores of 3D sorbent materials such as aerogels are filled with air. However, during the sorption of oil, the expulsion of air transforms air-filled aerogels into oil-filled ones, wherein the pores are occupied by oil^48,49^. Due to their low density, the absorption process of guar gum-based aerogels takes place entirely from the bottom (Fig. 7b). Furthermore, physical absorption is a cost-effective method for remediating oily water as it recovers sorbents more efficiently than chemical absorption. Absorption studies were conducted using a range of organic solvents and oils to gain further insight into the absorption properties of OTDS-modified GG/EL aerogel. The results revealed that OTDS-modified GG/EL aerogel exhibited efficient absorption capability when exposed to various organic solvents, with an absorption capacity from 20.64 to 45.1 g/g (Fig. 7c).

The recyclability of OTDS-modified GG/EL aerogel was evaluated using the squeezing method in the 10th cycle, as depicted in Fig. 7d. The resulted decrease in absorption capacity after the first cycle indicates that some of the pores in the aerogel collapsed permanently during the absorption and desorption process, resulting an absorption capacity reduction in the next subsequent cycles. However, the absorption capacity which is almost fixed in the subsequent cycles, confirms a suitable and desired reusability and durability of the achieved composite. In comparison with the other typical absorption materials, the natural-based aerogel synthesized from abundant biomass and guar gum (as a natural polymer), exhibited remarkable capacity for absorbing organic media (oily phases), as it listed in Table 1. Based on the tabulated data, it can be inferred that a reusable and durable composite aerogel has been successfully fabricated. As the other obtained and/or proposed green chemical strategic benefit, it can be expressed that the utilization of natural biomass in aerogel production can promote (develop) both affordability and sustainability. Reasonably, the prepared scalable OTDS-modified GG/EL aerogel is predicted to possess vast potential for application in the oily water (or oily wastewater) treatment in the near future.

**Table 1:** Comparison of the properties of several sorbent materials for oil removal.

Absorption materials	Absorption capacity (g/g)	WCA	Refs.
Chitosan/cellulose	13.77–28.20	152.8°	^50^
Modified carbon aerogel (MCA)	49.62 (diesel)	More than 90°	^51^
Lignin-based carbon aerogel	32–34	150°	^8^
SA/GO/SiO_2_-M	17.92–43.9	154°	^52^
Alginate–silica composite aerogels from rice husk ash	6–14	127°	^53^
Polystyrene foam	75	142°	^54^
Ambiently dried TEOS based silica aerogels	10–12.91	–	^55^
Epoxy-thiol-based silica aerogels	1694.9 mg/g (methylene blue dye)	–	^56^
OTDS-modified GG/EL aerogel	20.64–45.1	135°	This work

## Conclusion

This study presents a simple and efficient method for the preparation of a hydrophobic composite aerogel consisting of guar gum-esterified lignin. The preparation technique involves freeze-drying and chemical vapor deposition (CVD) hydrophobic modification. The resulting composite aerogel was thoroughly characterized using thermogravimetric analysis (TGA), scanning electron microscopy (SEM), and Fourier-transform infrared (FTIR) spectroscopy.

- The primary focus of the investigation was to explore the potential applications of lignin, particularly in oil absorption. The findings demonstrate that the incorporation of esterified lignin enhances the roughness and hydrophobicity of the aerogel, thereby improving its selectivity for the oil phase. The esterification of lignin was confirmed through comprehensive analyses, including FTIR, ^1^H NMR, and thermal analysis.
- SEM micrographs revealed the high porosity of the composite aerogel, enabling rapid oil absorption into its voids and validating the formation of uniform external and internal pore structures. This study highlights the significance of utilizing lignin, a byproduct of the pulp industry obtained through precipitation from black liquor, in the aerogel fabrication process. FTIR and TGA analyzes also were used to investigate the properties of the synthesized guar gum/esterified lignin aerogel.
- The study highlighted the importance of utilizing lignin, a waste product from the pulp industry, in the aerogel fabrication process.This approach offers a sustainable way to valorize waste material while reducing environmental impact.
- Furthermore, the fabricated composite aerogel exhibits excellent compressibility and recyclability, meeting the requirements for effective recycling of oil pollutants. These aerogels are regarded as environmentally friendly and cost-effective, with an uncomplicated preparation process.

## Data Availability

All data generated and/or analysed during the current study available from the corresponding author upon reasonable request.
